# Effect of cantilever length and alloy framework on the stress distribution in peri-implant area of cantilevered implant-supported fixed partial dentures

**DOI:** 10.1590/1678-775720150297

**Published:** 2016

**Authors:** Valdey SUEDAM, Rafael Tobias MORETTI, Edson Antonio Capello SOUSA, José Henrique RUBO

**Affiliations:** 1 - Universidade do Sagrado Coração, Faculdade de Odontologia, Bauru, SP, Brazil.; 2 - Universidade Federal de Alfenas, Faculdade de Odontologia, Departamento de Clínica e Cirurgia, Alfenas, MG, Brazil.; 3 - Universidade Estadual Paulista, Faculdade de Engenharia, Departamento de Engenharia Mecânica, Bauru, SP, Brazil.; 4 - Universidade de São Paulo, Faculdade de Odontologia de Bauru, Departamento de Prótese, Bauru, SP, Brazil.

**Keywords:** Implant-supported dental prosthesis, Dental implants, *In vitro* techniques, Mechanical stress

## Abstract

Because many mechanical variables are present in the oral cavity, the proper load transfer between the prosthesis and the bone is important for treatment planning and for the longevity of the implant-supported fixed partial denture.

**Objectives:**

To verify the stress generated on the peri-implant area of cantilevered implant-supported fixed partial dentures and the potential effects of such variable.

**Material and Methods:**

A U-shaped polyurethane model simulating the mandibular bone containing two implants (Ø 3.75 mm) was used. Six groups were formed according to the alloy’s framework (CoCr or PdAg) and the point of load application (5 mm, 10 mm and 15 mm of cantilever arm). A 300 N load was applied in pre-determined reference points. The tension generated on the mesial, lingual, distal and buccal sides of the peri-implant regions was assessed using strain gauges.

**Results:**

Two-way ANOVA and Tukey statistical tests were applied showing significant differences (p<0.05) between the groups. Pearson correlation test (p<0.05) was applied showing positive correlations between the increase of the cantilever arm and the deformation of the peri-implant area.

**Conclusions:**

This report demonstrated the CoCr alloy shows larger compression values compared to the PdAg alloy for the same distances of cantilever. The point of load application influences the deformation on the peri-implant area, increasing in accordance with the increase of the lever arm.

## INTRODUCTION

In fixed implant-supported prosthesis, the load applied to the occlusal surface of artificial teeth is transmitted along the framework and the abutment to the surrounding bone where most part of it is absorbed at the expenses of bone deformation.

According to Frost^8^ (2004), the bone reacts to forces according to the intensity of the tension. Bone responses to tension could then be divided in four intervals or *windows*: 1: *The acute disuse window* with tensions below 50 me (micrometer), resulting in bone loss because of an increase in the remodeling process; 2: *The adaptation window* with tensions between 50 me and 1500 me where physiological adaptation occurs with a balance between resorption and formation; 3: *The mild overload window* with tensions between 1500 me and 4000 me and where an increase in the modeling process occurs, improving bone structure; and 4: *The pathologic overload window* characterized by tensions above 4000 µε when bone resorption takes place.

According to Chang, et al.^6^ (2013), knowledge regarding the response of the peri-implant bone when the dental implant is excessively loaded is limited and the level of evidence is poor. With animal experimental studies showing conflicting results, it is unclear whether occlusal overload might cause marginal bone loss or total loss of osseointegration to already osseointegrated dental implants when the applied load exceeds the biologically-acceptable limit. This biological limit is also unknown. Furthermore, higher remodeling activity of the peri-implant bone is found around implants subjected to high loading forces. The strain values that can actually cause biological changes are not completely known^30^. Certain hormones and biochemical agents can also change the system, causing changes to the limits of tolerance^8^.

The implant-supported fixed prosthesis with distally extended lever arms present peculiar characteristics of force distribution since all the force applied in the posterior region of the cantilever is transmitted to the implants and consequently to the adjacent bone^29^. The findings of other studies, such as Benzing, et al.^5^ (1995) and Lewinstein, et al.^16^ (1995), demonstrate that the increase of the cantilever arm promotes an increase in stress concentration around the terminal implant. A cantilever arm of 10-20 mm is considered acceptable depending on the quality of the bone where implants are placed^14^ ^,^ ^20^ ^,^ ^21^ ^,^ ^27^.

According to Benzing, et al.^5^ (1995), the load application on the cantilever arm of an implant-supported framework produces deformation energy in the system that causes bending, depending on the differences of elastic modulus of several materials and components. Studies have demonstrated that the pattern of stress distribution among abutments depends, among other factors, on the alloy type used for framework^2^ ^,^ ^10^ ^,^ ^11^. According to some authors, Benzing, et al.^5^ (1995), Geng, et al.^9^ (2001) and Duyck & Naert^7^ (2002), a material with smaller elastic modulus offers smaller flexure resistance; frameworks made with rigid basic alloys suffer less deformation, being less prone to fatigue and, consequently, not overloading the screws. Some clinical^12^ and laboratory^1^ ^,^ ^13^ ^,^ ^23^ ^,^ ^26^ ^,^ ^29^ studies have used CoCr alloys for implant-supported prostheses frameworks.

The clinical success of osseointegrated implants are largely influenced by the manner mechanical stresses are transferred from the implant to the surrounding bone without generating forces of a magnitude that would jeopardize the longevity of implants and prostheses^25^. The force applied on the cantilevered implant supported fixed prostheses is transmitted to the peri-implant area. However, the magnitudes of the resultant stresses, considering the elasticity of the bone, are underestimated. The aim of this *in vitro* study was to verify the mechanical stress generated on the peri-implant bone of an implant prosthodontic system when: (1) a load is applied at different cantilever lengths and (2) alloys of different elastic modulus (E) are used to fabricate the framework.

## MATERIAL AND METHODS

A “U” shaped polyurethane model (PU, Axson – Cergy, St. Ouen l’Aumône, France) with the following dimensions: 100 mm in length, 13 mm in width, 19 mm in height, 46 mm in internal diameter, and 59 mm in external diameter was used to simulate the mandibular bone^18^ ^,^ ^19^. Two external hexagon Brånemark System^®^ Mk III Groov (Nobel Biocare – Göteborg, Västra Götaland, Sweden) implants of 3.75 mm in diameter and 13 mm in length were embedded in the model during polyurethane’s liquid pouring in a matrix. After polyurethane hardening, two multi-unit abutments (Nobel Biocare – Göteborg, Västra Götaland, Sweden) of 5 mm in length were manually screwed into the implants. A previously calibrated electronic torque controller device (Nobel Biocare Torque Controllerä, Göteborg, Västra Götaland, Sweden) was used to tighten the abutment screws to 20 Ncm torque.

**Figure 1:**
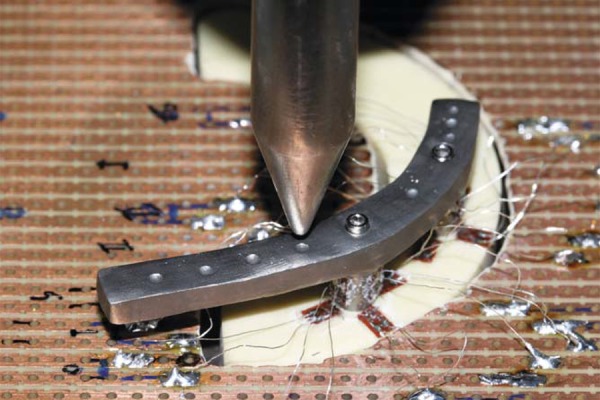
Positioning of the loading application point for application of the static 300 N load in the framework

Eight strain gages (KFG-02-120-C1-11, Strain Gages – Kyowa Electronic Instruments Co., Ltd., Tokyo, Honshu, Japan) were bonded with cyanoacrylate on the surface of the polyurethane model on the distal (D), lingual (L), mesial (M), and buccal (B) sides of implant 1 (distal) and implant 2 (mesial), as can be seen in Figure 1. Strain gauges are able to measure the tension suffered by an object or structure with which it is in close contact. The tension (ε) represents the amount of deformation of a body when submitted to a given force that can be tensile (+) or compressive (-).

The strain gauges were connected to a data acquisition device (NIcDAQ-9172 – National Instruments Corp., Austin, Texas, USA) that sent a signal to a LabVIEW 8.1 program for Windows (National Instruments Corp., Austin, Texas, USA) installed in a computer were inputs from the eight strain gauges were analyzed.

Two frameworks simulating a cantilevered implant-supported fixed partial dentures made of different alloys (CoCr and PdAg) were used in the study. The implants in the PU model were transferred and a gypsum model (Durone IV –Dentsply Ind. and Com., Petrópolis, RJ, Brazil) was obtained. Prosthetic cylinders were attached to the abutment replicas to construct an acrylic resin pattern (Durallay – Reliance Dental Mfg. Co., Alsip, Illinois, USA) with the following dimensions: 55 mm in length, 4 mm in width, and 4 mm in height. The cantilever arm measured 27 mm on the distal side of the bars. A silicon matrix helped to keep the same dimensions for all frameworks. The framework patterns were cast in one piece, one in cobalt-chromium alloy (Rexillium^®^ N.B.F. – Jeneric^®^/Pentron^®^ Incorporated, Wallingford, Connecticut, USA) cast on cobalt-chromium abutments and one in palladium-silver alloy (Pors-on 4 – Degussa S.A., São Paulo, SP, Brazil) cast on palladium-silver abutments. To allow the correct positioning of the loading application point, a dimple was made on the upper side of the framework at 5 mm, 10 mm and 15 mm distal to the center of the terminal abutment.

The frameworks were positioned in the PU model abutments and tested manually. As observed, only frameworks that adapted well to the abutments were to be approved for the tests. The two frameworks met this criterion and therefore there was no need to repeat the casts. Thus, titanium screws were tightened to 10 Ncm using an electronic torque controller (Nobel Biocare Torque Controllerä, Göteborg, Västra Götaland, Sweden).

The PU model was adapted and stabilized in a cylindrical steel base. The use of this rigid metallic base aimed at not interfering with the deformation of the PU model and not absorbing the load applied during the tests. Six test groups were formed (CoCr-5mm, PdAg-5mm, CoCr-10mm, PdAg-10mm, CoCr-15mm and PdAg-15mm), according to the alloy framework and to the point of load application.

Test specimens were taken to a Universal Testing Machine (model K-2000 MP – Kratos Equipamentos Industriais Ltda., São Paulo, SP, Brazil) and baseline readings of the absolute specific deformation values developed on each strain gauge were carried out prior to the load application (reading precision of order 1X10^-^ ^6^). Before initiating the readings on the deformation caused by loading the frameworks, the output of the measuring system was set to zero to separate from the deformation caused by abutment/prosthetic screw tightening. A round steel point was fixed to the load cell and adjusted to the pre-determined reference point in the framework (Figure 1). Thus, the testing machine was set to compression at a cross-head speed of 0.5 mm/min until it reached 300 N and stopped for one minute. The 300 N load was used to run the test according to the maximal occlusal bite force values found by Akça, et al.^3^ (2006) for implant-supported prostheses in opposition to natural teeth.

Deformation readings were taken at each one of the eight strain gauges for the duration of load application and 1 minute after load stabilization. Only the last 30 values of deformation were taken into account to ensure the maximum and stable levels of deformation were recorded for each site. Load application was repeated 5 times to calculate the mean and the standard deviation.

The two-way ANOVA statistical test was applied with the first variable being the type of alloy (CoCr and PdAg) and the second variable being the peri-implant region (D, L, M and B), which confirmed the presence of statistically significant differences. The Tukey test was applied to compare groups regarding the effect of two types of alloy. There was a statistically significant difference in each peri-implant region (p<0.05) for the force applied at the 5 mm cantilever (D1, M1, B1, D2 and B2), the 10 mm cantilever (D1, L1, M1, B1, D2, L2 and M2) and the 15 mm cantilever (D1, L1, M1, B1, D2, L2 and M2). The Pearson correlation test was applied to correlate the distance of load application on the cantilever and the values of deformation in each peri-implant region.

## RESULTS

**Figure 2:**
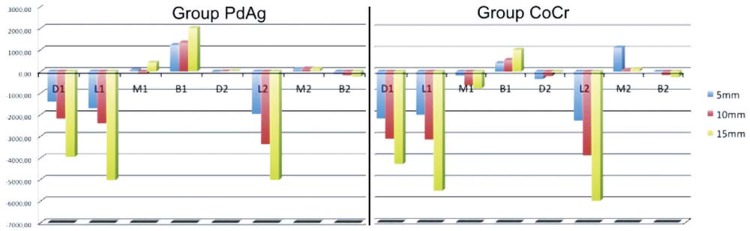
Graphic of the deformation means captured by the strain gauges in CoCr and PdAg alloy groups

The final mean deformation and the standard deviation values in each strain gauge are the result of 150 deformation readings. The numerical values obtained are expressed as tension (positive values) and compression (negative values), as seen in Table 1 and represented in Figure 2.

**Table 1:** Final mean and standard deviation of deformation values for each strain gauge with CoCr and PdAg alloys framework in tree conditions of load application (in mɛ)

	Implants	1				2			
	Stain gauges	D1	L1	M1	B1	D2	L2	M2	B2
	5 mm	-2181.89	-2004.14	-178.39	398.77	-343.7	-2276.29	1118.65	-17.51
Group CoCr		±215.48	±459.63	±72.39	±99.60	±109.99	±485.69	±1874.19	±66.37
	10 mm	-3113.64	-3160.51	-633.56	547.47	-215.146	-3885.74	90.39	-163.74
		±70.00	±93.10	±77.28	±7.08	±65.30	±240.98	±9.50	±53.17
	15 mm	-4302.05	-5538.95	-773.88	1018.86	-43.17	-6003.71	120.29	-252.24
		±81.58	±101.36	±80.82	±114.05	±26.09	±250.94	±9.80	±46.24
	5 mm	-1397.19	-1704.65	56.28	1231.87	-33.97	-1963.47	118.02	-112.62
Group PdAg		±35.68	±119.33	±14.21	±14.56	±12.11	±56.90	±39.03	±16.90
	10 mm	-2180.87	-2400.27	-102.24	1351.35	11.29	-3364.77	138.38	-183.15
		±62.86	±113.45	±13.25	±83.30	±33.72	±139.45	±8.36	±9.34
	15 mm	-3960.32	-5034.83	419.63	2009.83	25.42	-5019.2	127.31	-249.93
		±56.65	±271.49	±47.96	±52.13	±7.56	±312.71	±16.73	±16.01

With the load applied on the cantilever for all six groups, the most relevant results of compression occurred on the distal (D1) and lingual (L1) sides of implant I (distal), and on the lingual (L2) side of implant 2 (mesial). Tension occurred on the buccal (B1) side of implant I.

According to Suedam, et al.^29^ (2009), we can not sum the deformation suffered in every peri-implant region of each implant and consider this value as deformation of the entirety because each component of the system prosthesis/abutment/implant/bone can be found under various conditions of adaptation and load. As a result, a quantitative and qualitative evaluation of the results based on the statistical tests becomes necessary, which give us a biomechanical behavior view of the entire system involved with, and not only of the strain gauges or of the peri-implant regions individually.

The results of the Tukey test (Table 2) demonstrated the difference in the framework’s elastic modulus influenced the intensity of deformation occurred in the peri-implant region, as can be noted in Table 1 and in Figure 2. The Pearson correlation test showed positive correlation (Table 2).

**Table 2:** Tukey test for comparisons between groups and Pearson correlation test (distance X deformation) for each group

	Implants	1				2			
	Stain gauges	D1	L1	M1	B1	D2	L2	M2	B2
CoCr x PdAg	5 mm	0.000247*	0.196314	0.000296*	0.000223*	0.000433*	0.190638	0.266985	0.014714*
	10 mm	0.000223*	0.000223*	0.000223*	0.000223*	0.000318*	0.003219*	0.000237*	0.445044
	15 mm	0.000260*	0.004769*	0.000223*	0.000223*	0.000660*	0.000754*	0.441627	0.9187
CoCr	DISTANCE x DEFORMATION	-0.9875	-0.967	-0.9232	0.9183	0.8741	-0.9772	-0.3776	-0.8797
	p value	0.000*	0.000*	0.000*	0.000*	0.000*	0.000*	0.165	0.000*
PdAg	DISTANCE x DEFORMATION	-0.9748	-0.9417	0.674	0.9182	0.767	-0.9887	0.1591	-0.9742
	p value	0.000*	0.000*	0.006*	0.000*	0.001*	0.000*	0.571	0.000*

* statistically significant difference for p<0.05

## DISCUSSION

Knowledge on the amount of mechanical stress generated in the peri-implant area when load is applied along the cantilever arm is essential for the planning, execution and longevity of the treatment with implant-supported prostheses.

This study showed the pattern of bone deformation generated by applying a static force of 300 N varied according to: 1- The position where the strain gages were located on the peri-implant region (D1, L1, M1, B1, D2, L2, M2 and B2); 2- The point of load application (with 5 mm, 10 mm and 15 mm cantilevers); 3- Implant position relative to load application (I1 and I2); 4- Type of alloy used for making frameworks (CoCr and PdAg).

For all studied groups the behavior was singular, with tension forces present in a larger degree in the strain gauge located at the buccal region (B1) of implant 1, and compressive forces present in a larger degree in strain gauges at the distal and lingual region (D1, L1) of implant 1. This behavior can be due to the curved shape of the framework, where the resulting force tends to rotate the whole system to the distal and buccal sides.

The mean and standard deviation was calculated after five load applications for each group, result of 150 partial mean. After each load application the mechanical behavior of the each component of the system prosthesis/abutment/implant/bone suffered deformation under stress. This condition, associated to different degrees of fit among components, can be a possible cause of the high values of standard deviations found in this study.

According to data from other experiments^4^ ^,^ ^5^ ^,^ ^13^ ^,^ ^21^ ^,^ ^23^ ^,^ ^28^ ^,^ ^29^ in cantilevered prostheses, the most distal implants represent the fulcrum and, therefore, are subjected to compression forces while intermediary abutments suffer tension. In this study, the peri-implant regions were divided in four (D, L, M and B) allowing the observation that the distal and lingual sides of the most distal implant (I1) were subject to higher values of compression forces and that these values increased as the cantilever increased, as verified by the Pearson correlation test (Table 2).

The numerical values expressed in tension and compression for both alloys are the result of framework behavior due to load application, where the alloy elastic modulus influences the type of deformation and consequently the tension transmitted to the bone^2^ ^,^ ^10^ ^,^ ^11^ ^,^ ^13^ ^,^ ^17^ ^,^ ^29^. Because of the lower elastic modulus of the palladium-silver alloy compared to the cobalt-chromium alloy, and consequently for presenting a smaller flexion resistance, the results expressed in Table 1, and in Figure 2 demonstrated that when the load was applied to the CoCr alloy groups, larger compression values were recorded compared to PdAg alloy groups, for the same distances of cantilever. According to Rubo & Souza^23^ (2009) and Suedam, et al.^29^ (2009), the PdAg alloy deflects more, absorbing part of the load applied to the cantilever resulting compression forces of lower intensity being transmitted to the surrounding bone. On the other hand, because of its greater deflection when compared to CoCr alloy, the PdAg alloy presented the largest values of tension on the buccal side of implant 1 (I1), as confirmed by the Tukey test.

The load is transmitted to the surrounding bone where the most part of it is absorbed at the expense of deformation in bone structure, which is the less rigid structure in the system. Physiologic levels of tension serve also the purpose of bone remodeling. This mechanism would help maintain bone structural integrity indefinitely^22^. Nevertheless, mechanical overload can lead to biological failure^24^. When a pathological overload is applied to an osseointegrated implant, tension exceeds the physiological threshold tolerated by the bone and micro fractures may occur at the implant-bone interface. Repeated overload can lead to fatigue failure of the implant-bone interface, reducing peri-implant bone density and leading to the formation of bone defects such as craters. The pathologic overload window of Frost’s Theory represents this situation, when bone undergoes tensions above 4000 µε, being prone to resorption. It is important to note that inflamed peri-implant tissue reacts differently to occlusal overload promoting increased bone resorption, as demonstrated by Kozlovsky, et al.^15^ (2007).

The measure of tension generated in peri-implant area gave us the possibility of correlating these values with the bone remodeling theory^8^ in an attempt to clarify the biological process that takes place in that area, considering an ideal clinical condition. According to the polyurethane model validation studies made by Moretti, et al.^19^ (2011) and Miyashiro, et al.^18^ (2011), the homogeneity of polyurethane (PU) could favor its use in biomechanical studies of force distribution on implant supported prostheses, aimed at establishing correlations between strains generated in the peri-implant region and physiological strains as proposed by Frost’s Theory.

Nevertheless, it is known that considerable differences exist between this study and clinically integrated implants. Although polyurethane can present similar elastic modulus to bone, other features, such as anisotropy are difficult to mimic. This study does not claim that the strains found in the polyurethane model matches precisely to the *in-vivo* situation but acknowledges the biomechanical process of load transmission in an attempt to understand how bone tissue processes these transmitted loads.

**Figure 3:**
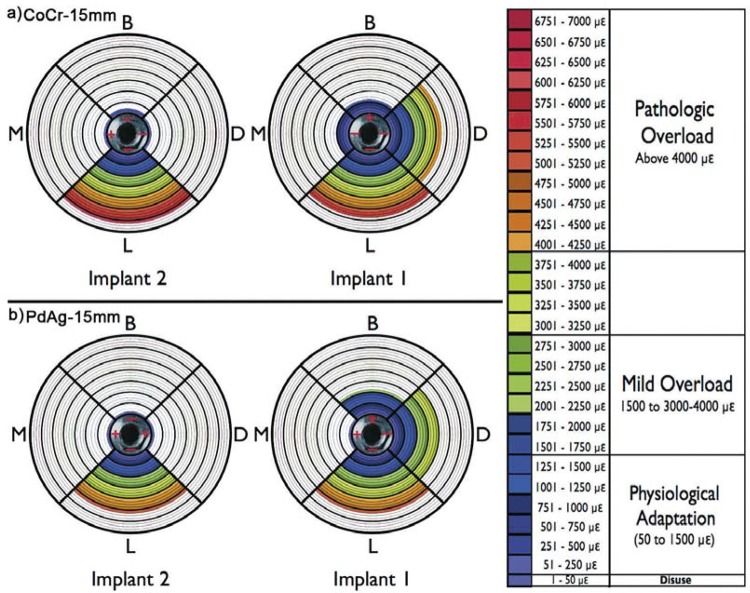
Strain diagrams with score label for group CoCr-15mm (a) and PdAg-15mm (b)

A strain diagram was used as a graphic representation of the deformation readings generated on each side of the peri-implant region. This diagram consists of a circular figure in a target shape with scales of 0 me to 7000 me, where readings of deformations generated on the distal, lingual, mesial and buccal sides of each implants of the groups CoCr-15 mm and PdAg-15 mm are visualized (Figure 3). In these diagrams, tensions above to 4000 µε can be seen for the CoCr-15 mm group (D1=-4302.05 me, L1=-5538.95 me); the same occurring with the PdAg-15 mm group (D1=-3960.32 me and L1=-5034.83 me). Based on the literature, these results have shown the two groups presented peri-implant regions within the pathologic overload window, being prone to bone resorption. According to this finding, cantilever arms smaller than 15 mm should be considered during the treatment planning of the mandibular implant-supported fixed partial dentures.

## CONCLUSIONS

Under the limited conditions of this *in vitro* study, the following conclusions were drawn: (1) The point of load application to the cantilever arm influenced the deformation of the peri-implant regions; (2) The type of alloy used for fabricating the framework influenced the biomechanical behavior and the deformations of the peri-implant regions; (3) Cantilever arms smaller than 15 mm must be considered for mandibular implant-supported fixed partial dentures.
